# Painful myokymia after surgery in a patient with Isaacs’ syndrome: a case report

**DOI:** 10.1186/s40981-020-00321-y

**Published:** 2020-02-15

**Authors:** Hiroai Okutani, Yukari Okano, Munetaka Hirose

**Affiliations:** Department of Anesthesiology and Pain Medicine, Hyogo College of Medicine, 1-1, Mukogawa-cho, Nishinomiya, Hyogo Japan

**Keywords:** Isaacs’ syndrome, Channel disease, Neuromuscular monitoring, Myokymia, General anesthesia, Epidural anesthesia, Case report

## Abstract

**Background:**

Isaacs’ syndrome is a peripheral nerve hyperexcitability syndrome and rare acquired channel disease. The symptoms (myokymia, neuromyotonia, and muscle spasm) emerge even during sleep. This report describes the anesthetic management, especially neuromuscular blocking drugs and postoperative pain, in a case of Isaacs’ syndrome.

**Case presentation:**

A 63-year-old woman with Isaacs’ syndrome underwent elective laparoscopic distal gastrectomy under general anesthesia without epidural anesthesia. She received double filtration plasmapheresis four times to alleviate symptoms before surgery. To avoid a prolonged neuromuscular blockade, we performed total intravenous anesthesia and titrated muscle relaxant with neuromuscular monitoring. Anesthetic management was performed without any problems. However, pain management after surgery proved difficult as she experienced severe pain due to myokymia.

**Conclusions:**

Despite attempts to minimize symptoms, severe postoperative pain associated with myokymia occurred. Continuous regional anesthesia should be considered to treat pain from abnormal discharge in the central nervous system in Isaacs’ syndrome.

## Background

Isaacs’ syndrome (acquired neuromyotonia) is a peripheral nerve hyperexcitability syndrome and rare acquired channel disease. Autoantibodies are produced to target presynaptic voltage-gated potassium channels (VGKCs). Suppressing the outward current of VGKC promotes neurotransmitter release and induces hyperexcitability in peripheral nerves. Symptoms emerge even during sleep, including myokymia (involuntary movement of muscles), neuromyotonia, muscle spasm, and muscle stiffness. Clinical presentation includes cramps, fasciculation, and myokymia.

Herein, we report the anesthetic management of a patient with Isaacs’ syndrome, detailing neuromuscular blocking drugs and symptoms of severe pain due to myokymia in the rectus abdominis muscle after laparoscopic gastrectomy. Informed written consent was obtained from the patient.

## Case presentation

A 63-year-old female (149 cm, 57 kg) was scheduled for elective laparoscopic distal gastrectomy under general anesthesia for gastric cancer. Her prior medical history included cerebral infarction and cervical spondylosis, controlled with medication. When she was 37 years old, she was diagnosed with Isaacs’ syndrome. Whole-body stiffness worsened, and double filtration plasmapheresis (DFPP) was performed four times per year to treat the symptoms.

The patient had previously received a surgical arteriovenous shunt in the left forearm, a subcutaneously fixed superficial artery, and an arteriovenous graft in the upper left arm; however, all had occluded within 1 year. Additionally, she had received a thrombectomy of the right femoral vein. As her blood vessels were easily occluded due to frequent vasospasms, a temporary cervical catheter had been used when providing DFPP treatment. The patient orally received tacrolimus (3 mg/day) to suppress the symptoms of Isaacs’ syndrome; dantrolene (150 mg/day), carbamazepine (600 mg/day), and gabapentin (900 mg/day) to relieve the muscle symptoms; dabigatran etexilate methanesulfonate (320 mg/day) as an anticoagulant; cilostazol (150 mg/day) to prevent cerebral infarction; and nicorandil (10 mg/day) as a vasodilator to suppress vasospasms.

The results of preoperative examinations were not remarkable except hemoglobin of 10.8 g/dL. The patient had myokymia of the bilateral upper extremities, neuromyotonia of the bilateral thumb, and left ptosis. We classified her physical status as American Society of Anesthesiologists physical status III. She was admitted to the hospital a month prior to the operation to receive DFPP four times.

When she arrived at the operating room, she was hemodynamically stable and treated with dantrolene (50 mg), carbamazepine (200 mg), gabapentin (300 mg), and nicorandil (5 mg) as premedications. We performed preoperative monitoring, including standard monitoring, neuromuscular monitor (TOF-Watch®), and electroencephalogram using the Bispectral Index monitor.

General anesthesia was induced with 100 μg fentanyl, target-controlled infusion of 2.5 μg/mL propofol, and 50 mg lidocaine after pre-oxygenation with 100% oxygen. After induction, we confirmed that T4/T1 was 100% using the TOF; we then administered 20 mg rocuronium. The time to get a TOF ratio of 0% was 4 min, and endotracheal intubation was carefully performed without complications. Anesthesia was maintained with oxygen, air, propofol (TCI concentration, 2.3 μg/mL), remifentanil (0.15–0.3 μg/kg/min), and intermittent intravenous administration of fentanyl. We also used the minimum amount of muscle relaxant. Rocuronium (10 mg) was administered when the TOF ratio increased to 50%. The total dose of rocuronium was 90 mg. Surgery proceeded uneventfully. For postoperative analgesia, infiltration anesthesia was performed at the rectus sheath with levobupivacaine (0.25%, 20 mL) and an intravenous fentanyl pump (0.5 μg/kg/h) started 1 h before the end of surgery. At the end of the procedure, we confirmed that the TOF ratio had recovered to > 90%. The residual neuromuscular block was antagonized using sugammadex (2 mg/kg), and propofol and remifentanil were discontinued. Shortly afterward, the TOF ratio recovered completely and spontaneous respiration resumed. The patient followed our commands and showed spontaneous breathing, and tracheal extubation was performed. The total operative time was 225 min and the anesthetic duration was 355 min. Subsequent to confirming stable vital signs and neuromuscular symptoms after extubation, the patient was moved to an intensive care unit.

After transfer, she complained about abdominal pain, so the infusion rate of fentanyl was increased to 1.0 μg/kg/h, and dexmedetomidine (0.4 μg/kg/h) was added to control pain. She was discharged to the general ward the day after surgery; however, the abdominal pain worsened because myokymia frequently occurs in the rectus abdominis muscle. The patient complained about severe pain (numerical rating scale, 8/10), so the infusion rate of fentanyl was increased to 2.0 μg/kg/h, which was effective for controlling pain. Afterward, she often experienced vomiting 8 days after surgery. The clinical course seemed to originate from postoperative pyloric stenosis. She received gastric bougie procedures two times after the surgery. The symptoms of Isaacs’ syndrome were stable after the postoperative pain was relieved, and she was discharged on postoperative day 66.

## Discussion

Isaacs’ syndrome (acquired neuromyotonia) is an extremely rare disorder. It is a peripheral nerve hyperexcitability syndrome and a type of acquired channel disease [1]. The immune system produces autoantibodies that target the presynaptic voltage-gated potassium channels, inducing hyperexcitability of peripheral nerves [1]. Involuntary movements emerge even during sleep and include myokymia, muscle relaxation disorder (neuromyotonia), muscle spasm, muscle stiffness, and hyperhidrosis. Since the first reports of this syndrome in 1961 [2], thymoma, myasthenia gravis, and anti-acetylcholine receptor disorder have been reported as complications of Isaacs’ syndrome [1, 3]. It was thought that this syndrome is an autoimmune disease because plasmapheresis is an effective method to treat the symptoms [4].

Typical examination reveals VGKC antibodies in the serum, myokymic discharge using electromyography, neuromyotonic discharge, and repeated low amplitude potential by nerve conduction study. Methods of treatment include administering antiepileptic drugs, muscle relaxants, and symptomatic treatment with analgesics. In cases of severe symptoms, patients are required to undergo steroid pulse therapy, immunoglobulin therapy, and plasmapheresis. As the abnormal discharge occurs at the neuromuscular junction, a proximal block might not suppress the discharge. In contrast, general anesthesia with neuromuscular blockade will abolish this discharge. Suppressing the abnormal muscle symptoms with only a local anesthetic agent would require considerably high concentrations. However, reports support the efficacy of muscle relaxants (depolarizing and non-depolarizing) and spinal and epidural anesthesia for suppressing spontaneous discharge [5, 6].

Since previous reports have shown an increase in acetylcholine release in these patients and tolerance of d-tubocurarine has been demonstrated in vitro, we can predict a tolerance for non-depolarizing muscle relaxants in patients with Isaacs’ syndrome [7, 8]. Some reports have suggested that general anesthesia in patients with Isaacs’ syndrome does not exacerbate neuromuscular disorders such as myotonia and involuntary muscular contraction [9–11]. However, respiratory complications caused by respiratory muscle weakness and laryngeal muscle myotonia can result from anesthesia [9–11]. Furthermore, patients with Isaacs’ syndrome are often comorbid with myasthenia gravis. In this case, the patient had strongly suspected complications with myasthenia gravis, and we had concerns about increasing the sensitivity of non-polarizing muscle relaxants. As a result, we performed general anesthesia using total intravenous anesthesia to avoid prolonged neuromuscular blockade [9]. She received DFPP four times before surgery, so the specific symptoms were improved, and the pharmacokinetics of muscle relaxant was almost normal. Therefore, the amount of muscular relaxant administered was normal, with minimal symptoms after surgery. However, neuromuscular monitoring is used to manage patients with neuromuscular diseases safely. It is critical to avoid the prolonged effects of muscle relaxants. We closely monitored the operative field; however, there were no complications during surgery. In addition, the surgeons also reported that there were no complications; there was also no bucking during the operation.

A few reports have shown that peripheral nerve block could be effective in controlling postoperative pain for patients with Isaacs’ syndrome [10, 11]. Ideally, epidural anesthesia is used to manage postoperative analgesia; however, in the present case, the patient was administered an antiplatelet drug and an anticoagulant due to a history of stroke and vasospasms. We did not provide epidural anesthesia due to these concerns and administered regional anesthesia instead. However, the patient had a sudden myokymia in the rectus abdominis muscle and suffered severe postoperative pain. Epidural anesthesia would likely be extremely effective in controlling myokymia and severe pain. When possible, we strongly recommend performing continuous regional anesthesia and early resumption of drugs to suppress the abnormal discharge in the central nervous system in Isaacs’ syndrome patients.

Here, we present a rare case of general anesthesia management in a patient with Isaacs’ syndrome. Attention should be paid to the control of muscle symptoms during the perioperative period, development of other complications, and administration of muscle relaxants. It is also effective to suppress the abnormal discharge in the central nervous system with spinal anesthesia or epidural anesthesia.

## Data Availability

Not applicable

## Abbreviations

DFPP: Double filtration plasmapheresis
TCI: Target-controlled infusion
TOF: Train-of-four
TOF-Watch®: Train-of-four monitor
VGKC: Voltage-gated potassium channels
